# Polymer Vesicles for Antimicrobial Applications

**DOI:** 10.3390/polym13172903

**Published:** 2021-08-28

**Authors:** Hui Sun, Yin Wang, Jiahui Song

**Affiliations:** 1 State Key Laboratory of High-Efficiency Coal Utilization and Green Chemical Engineering, Ningxia University, Yinchuan 750021, China; 2 School of Public Health and Management, Ningxia Medical University, Yinchuan 750004, China; wy9522@126.com; 3 Center of Scientific Technology, Ningxia Medical University, Yinchuan 750004, China; Songjh@nxmu.edu.cn

**Keywords:** polymer vesicle, self-assembly, polymer conjugates, antimicrobial, anti-biofilm

## Abstract

Polymer vesicles, hollow nanostructures with hydrophilic cavity and hydrophobic membrane, have shown significant potentials in biomedical applications including drug delivery, gene therapy, cancer theranostics, and so forth, due to their unique cell membrane-like structure. Incorporation with antibacterial active components like antimicrobial peptides, etc., polymer vesicles exhibited enhanced antimicrobial activity, extended circulation time, and reduced cell toxicity. Furthermore, antibacterial, and anticancer can be achieved simultaneously, opening a new avenue of the antimicrobial applications of polymer vesicles. This review seeks to highlight the state-of-the-art of antimicrobial polymer vesicles, including the design strategies and potential applications in the field of antibacterial. The structural features of polymer vesicles, preparation methods, and the combination principles with antimicrobial active components, as well as the advantages of antimicrobial polymer vesicles, will be discussed. Then, the diverse applications of antimicrobial polymer vesicles such as wide spectrum antibacterial, anti-biofilm, wound healing, and tissue engineering associated with their structure features are presented. Finally, future perspectives of polymer vesicles in the field of antibacterial is also proposed.

## 1. Introduction

Polymer self-assembly is a powerful tool to prepare functional nanomaterials with diverse morphologies, including micelles, cylinders, vesicles, nanobowls, flowers, and other highly ordered superstructures [1,2,3,4,5,6,7] which have shown great potentials in wide applications, including drug delivery, catalysis, energy storage, environment, and so forth [8,9,10,11,12,13,14,15]. Among the polymeric nanostructures, the polymer vesicle is a shining star due to its unique structure [16,17]. Typically, polymer vesicles are nanoscale hollow spheres composed of three parts, the interior holes, hydrophobic membranes, and hydrophilic coronas, which is similar to cell membranes that are composed of lipid bilayers but more stable and robust. The structural similarity of polymer vesicles and cell membranes motivates the scientists to reveal the secrets of life and mimic the functions of cells [18,19]. Therefore, the biomedical applications of polymer vesicles, including drug delivery, gene therapy, antibacterial, and cell mimicking, were of special interest to scientists [20,21,22,23].

The most studied building blocks of polymer vesicles are synthetic block copolymers with hydrophilic segments which form the coronas, and hydrophobic segments which form the membrane in a bilayer or interdigitated manner [17,24,25]. The manipulation of the membrane structure and the physiochemical property of the coronas of polymer vesicles is the focus of current studies [26,27]. For instance, the former determines the permeability of the membrane, in other words, regulating the load and on demand release of the cargoes [28,29], while the latter influences many factors in biomedical applications such as circulation time, cell toxicity, immune response, and so forth [27,30,31]. In addition, decoration of functional moieties onto the surface of polymer vesicles endowed them with specific but very important functions, including targeting ability and antibacterial activity [32,33].

Since the discovery of penicillin in 1928, antibiotics have played an important role in killing bacteria for decades. However, with the overuse and improper use of antibiotics, the emergence of bacterial drug resistance is becoming a severe problem. In particular, the multidrug-resistant (MDR) bacteria such as methicillin-resistant *Staphylococcus aureus* (MRSA) has threatened the health of human beings [34]. Non-antibiotic antimicrobial agents such as antimicrobial peptides (AMPs) have been used for a long time in the history of humans. AMPs represent a wide range of short, cationic peptides that can kill bacteria, which have been regarded as a promising solution to combat MDR bacteria [35,36,37]. Besides, other non-antibiotic antimicrobial agents, including silver ions and positively charged polymer-based nanostructures, have also been widely used in antibacterial fields [38,39,40,41]. Different from the sterilization mechanism of antibiotics, these non-antibiotic antimicrobial agents kill bacteria in a physical manner, such as destroying the membrane of bacteria, not involving the destruction of genetic materials, which avoids the generation of drug resistance [42,43,44].

**Figure 1:**
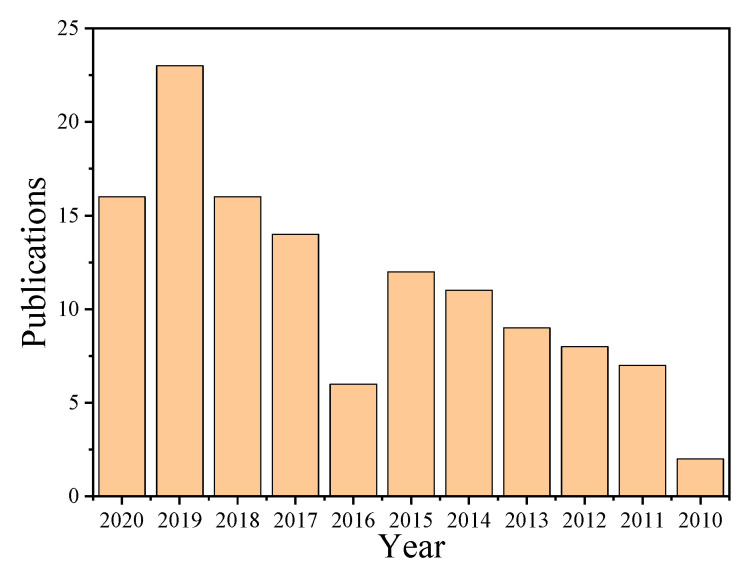
The publications per year of antibacterial polymer vesicles from 2010 to 2020 downloaded from Web of Science, searching with antibacterial and polymer vesicle as key words.

**Scheme 1:**
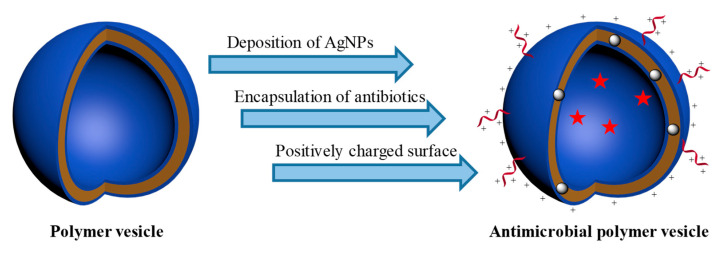
Design strategies of antimicrobial polymer vesicles.

Integrating antimicrobial agents and polymer vesicles can combine the advantages of both, which brings opportunities and new insights into the field of antimicrobial [45,46,47,48,49]. Though the investigation of antimicrobial polymer vesicles is still in its infancy, the publications per year have grown fast since 2010, indicating the rapid development of this field, as shown in Figure 1. We believe that the antimicrobial polymer vesicles will bring new opportunities in antibacterial and related biomedical applications. Typically, there are several strategies to integrate different antimicrobial agents with polymer vesicles, as shown in Scheme 1. For instance, silver nanoparticles (AgNPs) deposited on the membrane of polymer vesicles can endow the vesicles with excellent antimicrobial activity while preventing the agglomeration of AgNPs [38,50]. Antibiotics are usually encapsulated in the interior hole or the membrane of polymer vesicles to achieve the on-demand release at specific sites [51,52]. There are two strategies to introduce positive charges on the surface of polymer vesicles: functionalization of antimicrobial peptides and using positively charged segments with amino groups or quaternary amines as building blocks. The positively charged polymer vesicles have strong interaction with negatively charged bacterial cell membrane, which disturbs the local charge density and damages the stability of the cell membrane, leading to the flow out of the content of bacteria [53,54,55,56,57]. The advantages of integrating antimicrobial agents and polymer vesicles are as follows: (i) enhanced charge density on the surface of polymer vesicles, (ii) elongated circulation time in vivo, (iii) reduced cell toxicity due to the protection of the coronas of polymer vesicles, (iv) to accomplish on demand on/off of antimicrobial activity, and (v) to achieve antimicrobial and anticancer simultaneously.

## 2. Strategies to Integrate Polymer Vesicles with Antimicrobial Agents

### 2.1. Deposition of Silver Nanoparticles (AgNPs) onto the Membrane of Polymer Vesicles

AgNPs have been used as effective broad spectrum antibacterial agents to combat both Gram-positive and Gram-negative bacteria for a long time [58,59,60,61]. It is widely accepted that the silver ions released from the AgNPs are the antimicrobial active agents, which destroy the activity of proteases by penetrating into the membrane and cell walls of bacteria to induce the protein denaturation, leading to the death of bacteria [62,63,64,65]. It has been proven that the antimicrobial activity was determined by the diameter of AgNPs due to the speed of silver ions released from the AgNPs—the smaller the particle size, the stronger the antibacterial performance [66,67]. Therefore, the size control and stability of ultrathin AgNPs is the key to improve the antimicrobial activity of AgNPs.

Unfortunately, the ultrathin AgNPs are intended to aggregate to form larger particles to reduce the surface free energy, which reduced their antimicrobial activity sharply. Polymer vesicles are potential candidates to support AgNPs due to the facile designing of the physicochemical property of the coronas [39,68,69]. Traditionally, the silver ions are adsorbed onto the surface of polymer vesicles, followed by the in situ reduction to generate AgNPs. Therefore, the key to support AgNPs onto the membrane of polymer vesicles is to form a negatively charged surface to accumulate the silver ions. The hydrophilic and negatively charged carboxyl groups were usually introduced to the side chain of polymers, which were coated on the surface of the formed polymer vesicles [38,50,70]. For instance, Du and coworkers [38] synthesized an amphiphilic random copolymer poly(ethylene oxide)-*block*-poly(2-(dimethylamino)ethyl-*stat*-t-butyl acrylate) (PEO-*b*-P(DMA-*stat*-*t*BA)). After hydrolysis of *t*BA, acrylic acid (AA) segments with carboxyl groups were introduced to the side chain of the polymer, which then self-assembled into vesicles with a negatively charged surface. Followed by the adsorption and in situ reduction of silver ions, ultrathin AgNPs with a diameter of 1.9 ± 0.4 nm were deposited on the membrane of the vesicles, exhibiting excellent antibacterial activity against both Gram-negative and Gram-positive bacteria with quite low minimum inhibitory concentrations (MICs) of 16.9 and 8.45 μg mL^−1^, respectively.

**Figure 2:**
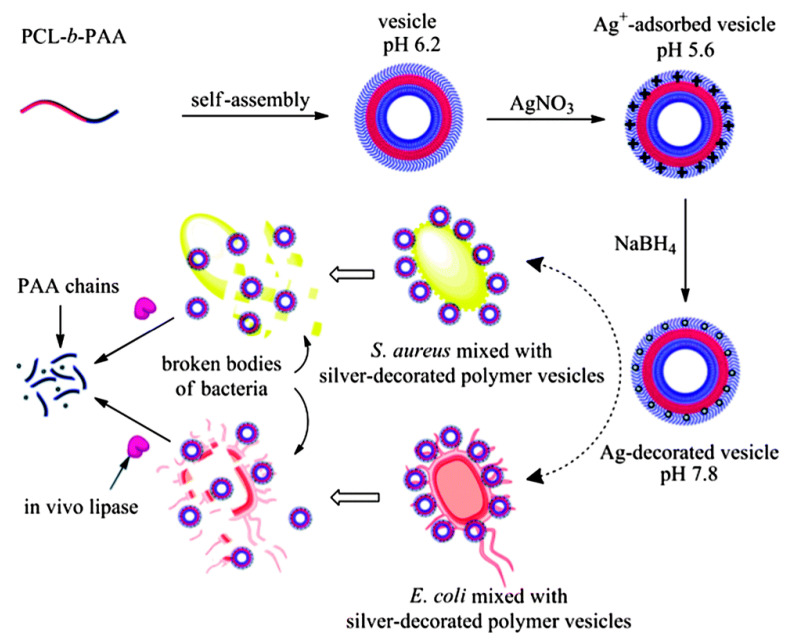
Schematic illustration of AgNPs-decorated biodegradable antibacterial polymer vesicles [50] (Reprinted with permission from [50]).

Later, the same group [50] prepared biodegradable polymer vesicles, with hydrophobic poly(*ε*-caprolactone) (PCL) forming the membrane and hydrophilic poly(acrylic acid) (PAA) forming the corona. AgNPs with diameter of 3.4 ± 1.2 nm were deposited on the membrane of the vesicles, as shown in Figure 2. The AgNPs decorated polymer vesicles displayed excellent antibacterial performance against both Gram-negative and Gram-positive bacteria, with MICs as low as 3.56 and 7.12 μg mL^−1^, respectively. Surprisingly, the AgNPs showed low cytotoxicity toward normal human liver L02 cells, and the cell viability maintained nearly 100% even after incubation for 48 h with AgNPs concentration of 20 μg mL^−1^, which is much higher than the MICs, demonstrating the high selectivity of AgNPs decorated polymer vesicles toward bacteria. The low cytotoxicity of AgNPs decorated polymer vesicles might be owing to the shield effect of the negative charges provided by PAA segments. Furthermore, the biocompatible PCL membrane can be biodegraded in the presence of *Pseudomonas* lipase that largely exists in pancreatic tissue, demonstrating the potential applications of AgNPs deposited biodegradable polymer vesicles in biomedicine.

### 2.2. Encapsulation of Antibiotics

Antibiotics have been the most effective antimicrobial agents to kill bacteria for a long time, which saved millions of people [44,71]. However, the emergence of drug resistance, especially MDR, has attracted people’s significant concerns [43,72,73]. The overuse and improper use of antibiotics caused the mutation and evolution of bacteria accumulated from the drug resistant genes within their plasmids that render drug treatment ineffective via drug altering enzymes, drug degrading enzymes, or drug efflux pumps [74]. Protection of the antibiotics from the destruction of enzymes pumped out of the cell membrane might be an effective strategy to reduce the drug resistance. Polymer vesicles are promising vehicles to deliver functional cargoes such as drugs, genes, DNAs, etc. to the cell nucleus, and antibiotics could also be directly transported to the nucleus of bacteria, preventing the destruction of enzymes and pumping out [51,68,75]. For instance, Battaglia et al. [51] reported the intracellular delivery of metronidazole or doxycycline to the *Porphyromonas gingivalis* infected oral epithelial cells by polymer vesicles, which were disassembled in the early endosomes due to the acidic condition, resulting in the release of metronidazole and doxycycline. Notably, the metronidazole or doxycycline loaded vesicles significantly reduced the number of intracellular *Porphyromonas gingivalis*, much better than free metronidazole or doxycycline. Besides, the metronidazole or doxycycline loaded vesicles also showed biocompatibility and low cytotoxicity to oral epithelial cells.

Convertine and coworkers [52] prepared pH responsive polymer vesicles that can efficiently encapsulate kanamycin to the *B. thailandensis* (AH183) infected RAW 264.7 cells. The low level of extracellular bacteria even after 24 h suggests that the polymersomes (also referred to as polymer vesicles) are able to effectively release the encapsulated antibiotic in a pH-dependent fashion while localized within intracellular compartments. In contrast to the cells treated with free kanamycin, the loaded responsive polymersomes showed very high levels of bacteria inhibition in both of the RAW cell lysates and the supernatants. These results suggested that the kanamycin was well encapsulated in the vesicles until the acid-triggered burst release inside the endosome of RAW cells.

**Figure 3:**
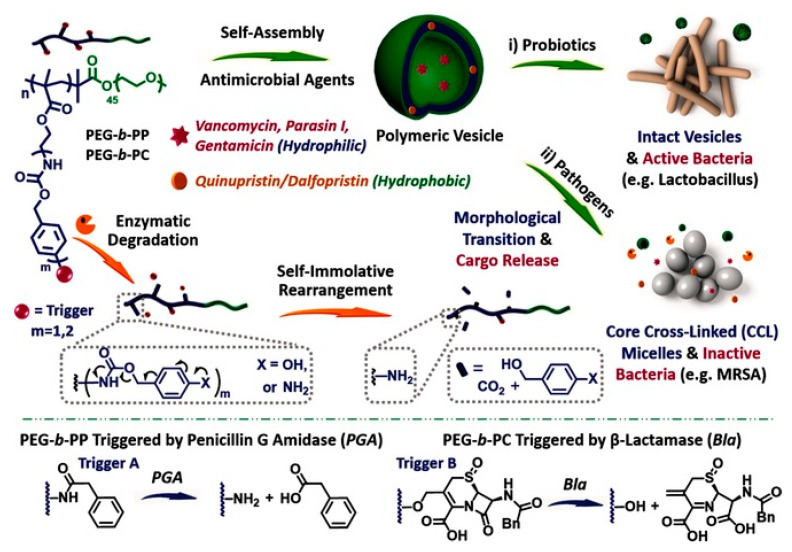
Enzyme-responsive polymer vesicles for bacterial strain-selective delivery of antibiotics [76] (Reprinted with permission from [76]).

Recently, Liu and coworkers [76] designed enzyme responsive polymer vesicles bearing self-immolative side chains for bacterial strain-selective delivery of antibiotics, as shown in Figure 3. The vesicles can encapsulate both of hydrophilic antibiotics, including vancomycin and gentamicin, and hydrophobic antibiotics such as quinupristin and dalfopristin, respectively, while the poly(ethylene oxide) (PEO) corona could reduce the cytotoxicity and improve the biocompatibility of the vesicles. Upon exposure to penicillin Gamidase (PGA) and *β*-lactamase (Bla), which are closely associated with drug-resistant bacterial strains, the self-immolative side chains were degraded, leading to the sustained release of antibiotics. In addition, considering that the Bla is the main cause of bacterial resistance to *β*-lactam antibiotic drugs, MRSA that could overexpress Bla was chosen to trigger the degradation of the vesicles and release of the encapsulated antibiotics, as well as evaluate the antimicrobial activity of the antibiotics loaded vesicles. Comparing with other bacterial stains such as *B. longum*, *L. acidophilus*, and *E. faecalis*, only MRSA was effectively inhibited due to the release of vancomycin induced by the degradation of the vesicles triggered by Bla, demonstrating the selective antimicrobial activity of the antibiotics-loaded vesicles.

### 2.3. Introduction of Positively Charged Coronas

The cell membranes of bacteria are negatively charged. Therefore, despite the use of antibiotics to destroy the genetic materials of bacteria, positively charged nanostructures usually showed antimicrobial activity due to the strong interaction with the cell membrane of bacteria [77,78,79,80,81]. Functionalization of positively charged molecules, such as AMPs and amines to the surface of polymer vesicles or using positively charged polymer chains as building blocks, are the main strategies to introduce positive charges to the surface of vesicles [82,83,84,85,86,87]. In this section, we will discuss the preparation of positively charged polymer vesicles with amino group contained polymers or functionalization with AMPs. And the preparation of positively charged vesicles using polymer–polypeptide conjugates will be discussed in the next section. Compared to their linear counterparts, polymeric aggregates including micelles, vesicles, etc. usually exhibited superior antimicrobial activity due to the enhanced local charge density [88,89].

**Figure 4:**
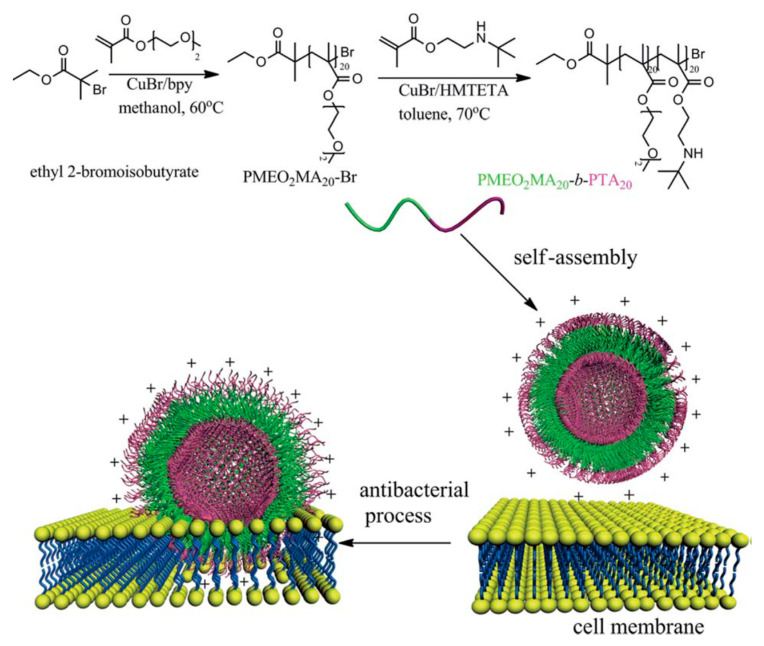
Polymer vesicles by direct dissolution of a diblock copolymer in water, exhibiting a superior antibacterial activity to their linear counterparts. Green: the thermo-responsive PMeO_2_MA block forms the vesicle membrane at 37 °C; purple: the antibacterial PTA block forms the vesicle corona [53] (Reprinted with permission from [53]).

Polymers with quaternary ammonium groups, especially their aggregates, usually nanoparticles have shown excellent antibacterial performance against both Gram-positive and Gram-negative bacteria [75,90,91]. However, the strong positive charges may lead to severe cytotoxicity such as hemolysis, which is a common side-effect of cationic polymers. Therefore, using polymeric building blocks with moderate positive charges to form nanostructures might be a comprehensive strategy to balance the antimicrobial activity and cytotoxicity. For instance, Du and coworkers [53] used poly [2-(2-methoxyethoxy)ethyl methacrylate]-*block*-poly[2-(tert-butylaminoethyl) methacrylate] (PMEO_2_MA-*b*-PTA) as building block to prepare cationic polymer vesicles, as shown in Figure 4. The water-soluble polymer could self-assemble into vesicles with PMEO_2_MA, which forms the membrane, and cationic PTA, which forms the corona due to the hydrophilic/hydrophobic transition of PMEO_2_MA at 37 °C. Comparing to the polymer chains, polymer vesicles exhibited much better antimicrobial efficacy against both Gram-negative and Gram-positive bacteria under physiological conditions due to the enhanced local charge density. Moreover, this polymer could also self-assemble into high-genus vesicles by solvent switch method, which exhibited better antibacterial activity than the simple vesicles due to the efficiently exposed surfaces [92]. It is noteworthy that the high-genus vesicles showed no cytotoxicity toward L02 liver cells when the concentration was lower than 1.0 mg mL^−1^. Besides, the red blood cell hemolysis experiment also indicated a high *H*_50_ (hemolysis rate of 50%) of 4.7 mg mL^−1^, demonstrating the excellent blood compatibility of the high-genus vesicles.

Decorating AMPs onto the surface of polymer vesicles or liposomes is another effective strategy to endow the vesicles with antimicrobial activity [75,84,89]. Recently, Webster et al. [70] prepared polymer vesicles by the self-assembly of PEG-*block*-poly(d,l-lactide) [PEG-*b*-PDLLA], and the linear AMP, cationic proline (49%) and arginine (24%)-riched porcine cathelicidin, PR-39 was decorated on the surface of the vesicles. The AMP PR-39 could not inhibit the proliferation of *S. aureus* and MRSA independently. However, after being linked onto the surface of the vesicles and synergized with AgNPs decorated on the membrane and methicillin antibiotics encapsulated in the cavity, the multifunctional vesicles displayed excellent inhibition of MRSA over 23 h with AgNPs concentration of 11.6 μg mL^−1^ and AMP concentration of 14.3 × 10^−6^ M, demonstrating the multifunctionality and potential of polymer vesicles in combating MDR bacteria.

### 2.4. Using Antibacterial Polypeptides as Building Blocks

**Figure 5:**
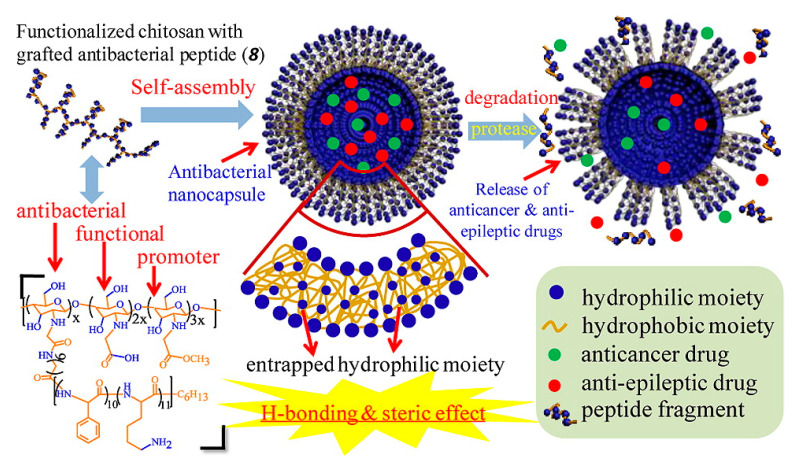
Antimicrobial polymer vesicles as drug carrier self-assembled from the antibacterial polypeptide-grafted chitosan [97] (Reprinted with permission from [97]).

Solid-phase synthesis and polymerization of α-amino acid-*n*-carboxyanhydride (NCA) monomers (NCA polymerization) were the most widely used methods to synthesize antimicrobial polypeptides [57,93,94,95]. The NCA polymerization was conducted in solution initiated by amino groups, which was facilitated in combination with functional polymers. The synthetic methods of antimicrobial polypeptide–polymer conjugates were summarized in our previous review [96]. Typically, a polymer with amino group terminals was used as macro-initiator to trigger the polymerization of NCA monomers, leading to the formation of polymer–polypeptide copolymers. The antimicrobial polypeptides segment is usually formed by the random polymer of positively charged and hydrophobic amino acids, which formed the corona of the vesicles to enhance the interaction with the cell membrane of bacteria. For instance, Du and coworkers [97] grafted the random co-polypeptide of phenylalanine (Phe) and lysine (Lys) onto the backbone of chitosan by NCA polymerization, as illustrated in Figure 5. The grafted copolymer could self-assemble into polymer vesicles with positively charged surfaces, which exhibited excellent antimicrobial activity with a MIC of 16 μg mL^−1^, much lower than its linear analogue (31 μg mL^−1^). In addition, the *H*_50_ of the vesicle and polymer chains were 700 and 110 μg mL^−1^, respectively, corresponding to a selectivity (the value of *H*_50_/MIC) toward bacteria and blood cells of 44 and 3.4, respectively, indicating the high antimicrobial activity and blood compatibility, as well as the great potential of the vesicles used in drug delivery while eliminating the inflammation at the focus of infection simultaneously.

Later, the same group conducted systematic studies using antimicrobial polypeptide–polymer conjugates as building blocks to prepare antimicrobial vesicles and investigate their biomedical applications [54,98,99,100,101]. For example, a folic acid labeled biodegradable PCL-polypeptide vesicle was prepared to inhibit bacteria and targeted drug delivery [54]. Moreover, they prepared penicillin loaded PCL-*b*-P(Lys-*stat*-Phe) vesicles embedded in the PEG/chitosan hydrogels to achieve the rapid and long-term antibacterial simultaneously, in which the encapsulated penicillin could kill bacteria rapidly while the sustained release of penicillin and the intrinsic antimicrobial activity of the vesicles could inhibit the growth of bacteria for a long time [99]. Very recently, they prepared dual corona vesicles by the co-assembly of two polymers with the same hydrophobic chain, and ciprofloxacin hydrochloride was encapsulated in the interior of the vesicles. Taking advantage of the antimicrobial activity of ciprofloxacin hydrochloride and the vesicles, as well as the shield effect of the dual coronas, the ciprofloxacin hydrochloride loaded dual corona vesicles exhibited excellent treatment effect to biofilm-induced periodontitis in vivo [102]. Those results demonstrated the potential of antimicrobial polymer vesicles in diverse applications in the field of biomedicine.

## 3. Antimicrobial Applications of Polymer Vesicles

### 3.1. Broad-Spectrum Antibacterial

Typically, Gram-negative bacteria *Escherichia coli* (*E. coli*) and Gram-positive bacteria *Staphylococcus aureus* (*S. aureus*) were used as representatives to evaluate the broad-spectrum antimicrobial activity of polymer vesicles. There are two widely used methods to determine the antimicrobial performance of polymer vesicles. One is to count the number of bacterial colonies incubated with different concentrations of polymer vesicles for a specific time, while the other is to monitor the optical density (e.g., at 600 nm) of the bacterial solution with the addition of vesicles by UV-vis spectrometer at different time. Du and coworkers reported pioneering studies of polymer vesicles in the application of antibacterial [38,88]. In general, the polymer vesicles with positively charged surface exhibited both Gram-positive and Gram-negative bacteria inhibition activity due to the non-selectivity of the physical damage of the cell membrane of bacteria [83,103,104,105]. Comparing to their linear counterparts, polymer vesicles usually showed better antimicrobial activity due to the enhanced local charge density and reduced cytotoxicity toward mammalian cell, owing to the shield effect of the coronas, which was described in the previous sections.

**Figure 6:**
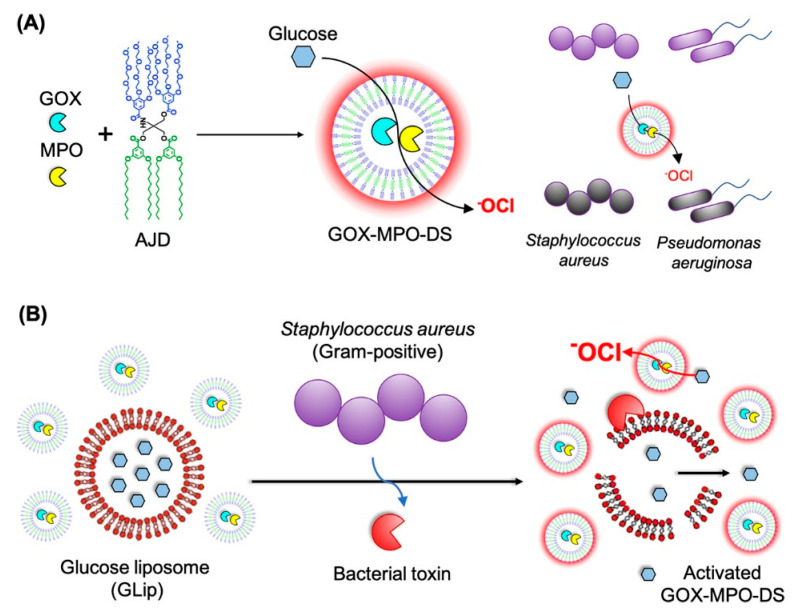
Preparation of GOX-MPO-DS and proposed bacteria-mediated switch-on mechanism: (**A**) Encapsulation of GOX and MPO within a vesicle composed of AJDs to produce the antibacterial nanoreactor GOX-MPO-DS, which produces ^−^OCl to kill Gram-positive *S. aureus* and Gram-negative *P. aeruginosa* upon addition of glucose and (**B**) Introduction of a bacterial switch-on mechanism enabled by the toxin-induced release of glucose from giant unilamellar vesicles [105] (Reprinted with permission from [105]).

Loading of bioactive enzymes by vesicles to generate antimicrobial active species is another effective method to combat bacteria. For example, Stevens and coworkers [105] prepared dendrimersomes by the co-self-assembly of amphiphilic Janus dendrimer (AJD), glucose oxidase (GOX), and myeloperoxidase (MPO) (noted as GOX-MPO-DSs), as illustrated in Figure 6A. The GOX and MPO were encapsulated in the cavity of the vesicles, and hypochlorite (^−^OCl) was produced with the addition of glucose, which is an effective antimicrobial species to kill both Gram-positive *S. aureus* and Gram-negative *P. aeruginosa*. Considering the high toxicity of ^−^OCl to mammalian cells, they developed a strategy to activate ^−^OCl production in a localized manner in response to a bacterial stimulus. The glucose encapsulated giant unilamellar vesicles were mixed with the GOX-MPO-DSs, and the release of the encapsulated glucose could be triggered by bacterial toxins, leading to the generation of ^−^OCl. In other words, the ^−^OCl was only generated in the presence of bacterial growth, as shown in Figure 6B. The antibacterial tests demonstrated the excellent antimicrobial activity (>99.9% elimination) of GOX-MPO-DSs against both of *S. aureus* and *P. aeruginosa* with a concentration of 1 × 10^12^ particles mL^−1^, concentrations of Cl^‒^ and glucose of 137 and 20 mM, respectively. Moreover, the GOX-MPO-DSs exhibited excellent cytocompatibility when incubated with the RAW 264.7 cell line without the trigger of bacteria.

### 3.2. Selective Antimicrobial and Anti-MDR Bacteria

Selective antibacterial could also be achieved by polymer vesicles [76,85,106]. For instance, Ghosh et al. synthesized polyurethanes with a primary and secondary amine group, named PU-1 and PU-2, which could self-assemble into capsules due to the intrachain hydrogen interaction in acid water [85]. The capsules showed selective interaction with bacterial cell mimicking liposomes over mammalian cell, mimicking liposomes attributed to the electrostatic interaction and hydrophobic effect. The antibacterial test demonstrated the ultralow MICs of 7.8 and 15.6 μg mL^−1^ for PU-1 and PU-2 against *E. coli* and over 500 μg mL^−1^ against *S. aureus*, respectively, indicating the selective killing of *E. coli* of polyurethane capsules. Besides, the *H*_50_ of the PU-1 and PU-2 were 453 and 847 μg mL^−1^, respectively, over 50 times higher than the MICs, implying the high selectivity of PU-1 and PU-2 to kill Gram-negative bacteria.

**Figure 7:**
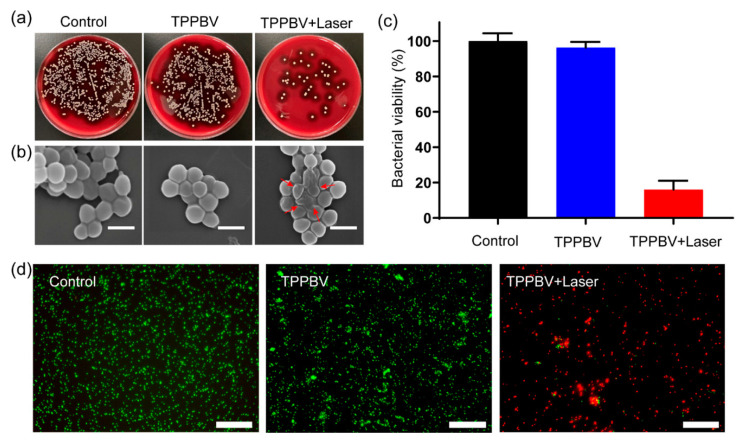
Antibacterial and anti-biofilm effect of photothermal vesicles in vitro. (**a**) Photographs of MRSA bacterial colonies treated with vesicles (500 μg mL^−1^), upon laser irradiation (635 nm, 600 mW cm^−2^) for 10 min and (**b**) corresponding SEM images of the MRSA. Scale bar, 1 μm. (**c**) Colony-forming unit (CFU) count of MRSA after various treatments. (**d**) Live/dead fluorescence staining merge pictures of the MRSA biofilm after treatment for 10 min. Scale bar, 75 μm [107] (Reprinted with permission from [107]).

Polymer vesicles combined with various antimicrobial active species are potential candidates to combat MDR bacteria [70,107,108]. For example, Webster and coworkers [70] prepared multifunctional polymer vesicles by decorating AMP, PR-39 on the surface, encapsulating methicillin in the cavity, and deposition of AgNPs on the membrane to combat MRSA. Recently, Zhou et al. [107] designed an alternating copolymer bearing zinc porphyrin pendants, which could self-assemble into polymer vesicles with high photothermal conversion efficiency up to 54.1%. The vesicle showed distinct antimicrobial activity to MRSA and extended-spectrum *β*-lactamases *Escherichia coli* (ESBL *E. coli*) upon laser irradiation. As illustrated in Figure 7a–c, 84.0% of MRSA colonies were inhibited after the laser irradiation with the temperature elevated to 62 °C. Meanwhile, the photothermal vesicles also revealed distinct inhibition efficiency of 83.7% for ESBL *E. coli*. In addition, the authors also investigated the anti-biofilm activity of the photothermal vesicles toward MRSA biofilm, as shown in Figure 7d. After treatment for 10 min with photothermal vesicles and irradiation, most of the MRSA were killed, demonstrating the outstanding anti-biofilm effect of the photothermal vesicles assisted with irradiation.

### 3.3. Antimicrobial Drug Carrier

Considering the unique structure of polymer vesicles with hydrophilic interior cavity and hydrophobic membrane, antimicrobial polymer vesicles are ideal candidates to encapsulate and deliver functional cargoes such as antibiotics, drugs, and other biomacromolecules to realize specific functions while maintaining the antimicrobial activity of the carriers [109]. The encapsulation of antibiotics to enhance the antimicrobial activity of polymer vesicles has been discussed in Section 2.2. This section mainly focuses on the use of antimicrobial polymer vesicles as drug carriers to deliver functional drugs, such as anticancer drugs. The reduction of the cytotoxicity or improvement of the selectivity of the antimicrobial vesicles toward bacteria and mammalian cells is the key to design antimicrobial carriers. Du and coworkers conducted pioneering investigations in this field and prepared vesicles by using antibacterial polypeptides as building blocks, which exhibited improved biocompatibility compared with their linear counterparts. Therefore, they proposed the new concept of an “armed” carrier to achieve the dual functions of antimicrobial and anticancer, which is described in Section 2.4 [92,97].

**Figure 8:**
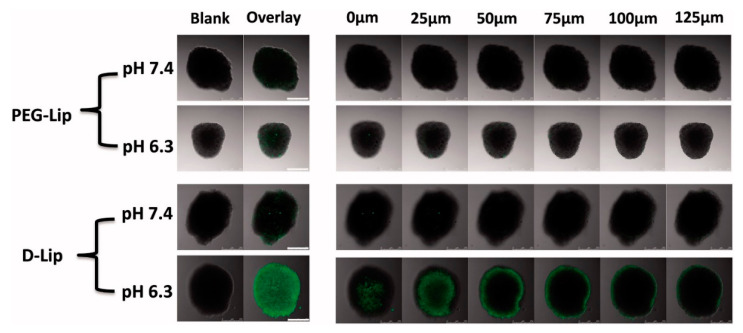
The uptake of CFPE-labeled D-Lip under both pH 7.4 and 6.3 within different depths into the tumor spheroid. The scale bars represent 250 μm [89] (Reprinted with permission from [89]).

In addition, despite the excellent antimicrobial performance, the AMPs functionalized polymer vesicles also exhibited enhanced cell uptake and tumor accumulation property. For instance, He and co-workers [89] functionalized a pH-responsive AMP, [D]-H_6_L_9_, on the surface of a polymer vesicle to construct the pH-responsive vesicle. The AMP is negatively charged at pH 7.4, making the vesicle nontoxic and serum protein resistant with long-term circulation in blood. When the pH is decreased to 6.3, the vesicle is positively charged owing to the protonation of histidines in the sequence of [D]-H_6_L_9_, leading to the enhanced cellular uptake and tumor spheroid uptake, as shown in Figure 8. Considering the acidic microenvironment at tumor sites, the AMP-modified vesicles can be selectively accumulated in tumors and taken up by cancer cells promoted by the positively charged surface. The macropinocytosis and caveolae-mediated endocytosis by tumor cells induced by the positively charged AMP on the surface of the vesicles facilitate the lysosomes escape of the AMP modified vesicle, promoting the targeting of anticancer drugs to the nucleus of cancer cells. Though the antimicrobial performance is not the focus of this study, we believe that the concept of this study may bring new insights into the field of antimicrobial and anticancer.

### 3.4. Anti-Biofilm, Wound Healing, and Tissue Engineering

Biofilms are aggregates of microorganisms, in which cells are frequently embedded in a self-produced matrix of extracellular polymeric substances (EPS) that are adherent to each other [110,111,112]. The bacteria density is very high in the biofilm and protected by the EPS from the destruction of external antimicrobial agents. Antimicrobial polymer vesicles have shown significant potentials in anti-biofilm, wound healing, and tissue engineering due to their enhanced antimicrobial activity, penetration capability, and multifunctionality [70,107,113]. For instance, Blackman and co-workers [86] prepared glucose oxidase-loaded poly(ethylene glycol)-*block*-poly(2-hydroxypropyl methacrylate) (PEG-*b*-PHPMA) vesicles by polymerization-induced self-assembly (PISA) and demonstrated that the vesicles can “switch on” their antimicrobial activity under the stimuli of glucose. Upon exposure to the circumstance with glucose, hydrogen peroxide was produced by the catalysis of glucose oxidase, which was highly toxic to bacteria. The polymer vesicles exhibited excellent antibacterial activity toward a range of Gram-negative and Gram-positive bacterial pathogens, including MRSA at high glucose concentrations, and comparable anti-biofilm activity against *S. aureus* clinical isolate biofilms.

**Figure 9:**
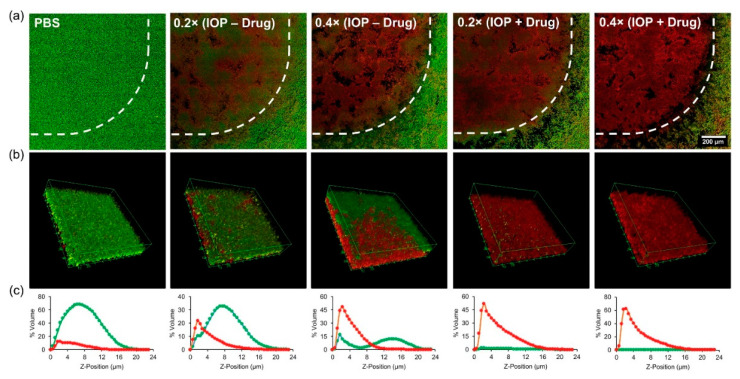
Anti-biofilm activity of iron oxide-encapsulated vesicles as a function of drug and SPION loading. (**a**) Tile scans of the Live (green) and dead (red) bacteria by laser scanning confocal microscopy after 24 h within the magnetic field (pointed by dash line) at different concentrations (1 × stock equals to 100 μg mL^−1^ IOP; 50 μg mL^−1^ methicillin). (**b**) Three-dimensional reconstruction of z-stacks of the biofilm. (**c**) The percentage of biofilm volume occupied by live and dead bacteria as a function of biofilm depth (0 μm = bottom) [111] (Reprinted with permission from [111]).

Webster et al. [111] prepared a biocompatible multi-compartment polymer vesicle with superparamagnetic iron oxide particles (IOPs) embedded in the membrane and methicillin encapsulated in the cavity to destroy biofilms. With the assistance of an external magnetic field, the IOPs-encapsulated vesicles could efficiently penetrate *Staphylococcus epidermidis* biofilms with a thickness of 20 μm. Laser scanning confocal microscopy revealed differential bacteria death as a function of drug and IONs loading, as shown in Figure 9a. The distinct boundaries pointed by the dash line indicated the importance of magnetic field to promote the anti-biofilm activity of the polymer vesicles. When the concentrations of IOP and methicillin were 40 and 20 μg mL^−1^, respectively, all of the bacteria were eliminated throughout the biofilm thickness, as illustrated in Figure 9b,c. Importantly, this formulation was selectively toxic towards methicillin-resistant biofilm cells rather than mammalian cells, demonstrating the high selectivity of the multi-compartment vesicles.

Recently, Du and coworkers [102] prepared dual corona polymer vesicles by the co-assembly of PEO-*b*-PCL and PCL-*b*-P(Lys-*stat*-Phe), which could efficiently deliver antibiotics to biofilms and treat biofilm-induced periodontitis at a much lower concentration of antibiotics due to the synergy of the intrinsic antimicrobial activity of the polymer vesicles and antibiotics. The dual corona was formed by PEO and P(Lys-*stat*-Phe) chains, and the former provided protein-repelling capability to penetrate the EPS, while the latter endowed the vesicles with positively charged surface and broad-spectrum antimicrobial activity. The dual corona polymer vesicles exhibited very high selectivity to kill bacteria with MICs of 128 μg mL^−1^ against both *E. coli* and *S. aureus*. However, the cell viability was still as high as 80% at a concentration of 100 μg mL^−1^. The dual corona vesicles could also deliver ciprofloxacin to the depth of *E. coli* and *S. aureus* biofilm efficiently, leading to a 50% reduction of the dosage of ciprofloxacin to inhibit the biofilm. Besides, in vivo experiments demonstrated that the ciprofloxacin-loaded dual corona vesicles could effectively alleviate inflammation and reduce dental plaque of a rat periodontitis model.

Taking advantage of the unique structural property of vesicles in drug delivery systems, the antimicrobial polymer vesicles also showed potential in tissue engineering and wound healing with the encapsulation of functional molecules [107,114,115]. For instance, Du and coworkers [114] designed a series of antibacterial peptide–mimetic alternating copolymers (PMACs) with different repeating units, and the PMAC with repeating unit of 14 exhibited the best antibacterial activity against both of *E. coli* and *S. aureus* with ultralow MICs of 8.0 μg mL^−1^. Notably, the PMACs could self-assemble into polymer vesicles in pure water while maintained the excellent antimicrobial activity. Growth factors could be encapsulated in the antimicrobial vesicles and released during the long-term antibacterial process to promote the repair of bones. In vivo experiments demonstrated the promoted bone repair capability of the growth factors-loaded vesicles in rabbit models compared with the control groups.

**Figure 10:**
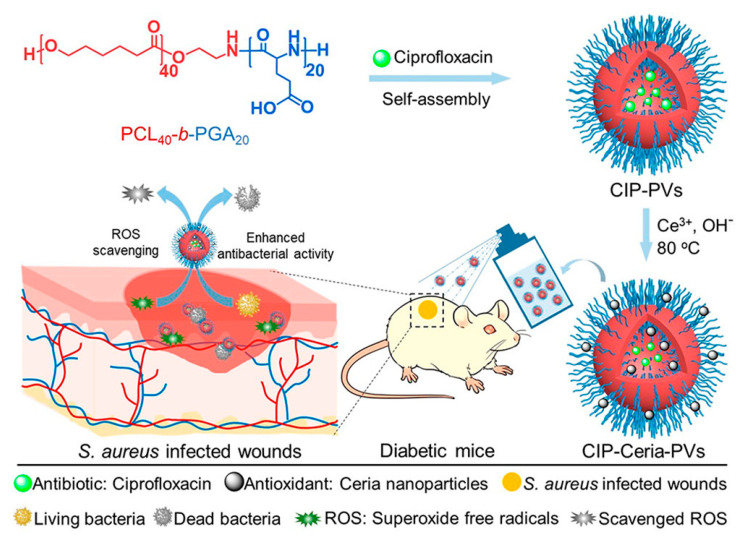
Illustration of the preparation of ciprofloxacin and ceria loaded polymer vesicles and the combined antioxidant–antibiotic treatment of infected diabetic wounds [115] (Reprinted with permission from [115]).

Very recently, the same group [115] designed antioxidant–antibiotic co-loaded polymer vesicle to cure the infected diabetic wounds, which usually caused a diabetic ulcer due to the high local concentration of reactive oxygen species (ROS). As illustrated in Figure 10, a biodegradable polymer PCL-*b*-PGA was designed to self-assemble into vesicles and encapsulate ciprofloxacin into the hydrophilic cavity. Ceria nanoparticles could be deposited on the corona of the vesicles due to the electrostatic interaction between Ce^3+^ ions and carboxyl groups. The ciprofloxacin and ceria nanoparticles co-loaded vesicle (CIP-Ceria-V) showed excellent antioxidant capability, in which 50% inhibition rate of superoxide free radicals was obtained at a very low concentration of ceria (1.25 μg mL^−1^). With the concentration of ceria increased to the range of 5 to 20 μg mL^−1^, the CIP-Ceria-V exhibited the best protective effect toward normal L02 cells, demonstrating the entirely elimination of superoxide free radicals. Besides, the CIP-Ceria-V also showed synergetic antimicrobial activity between ciprofloxacin and ceria nanoparticles, giving MICs of 0.0375 and 0.10 μg mL^−1^ against *E. coli* and *S. aureus*, only half of the free ciprofloxacin. In vivo experiments on diabetic mice further demonstrated the excellent antioxidant and antimicrobial activity of CIP-Ceria-V to promote the healing of the *S. aureus* infected diabetic wounds with 14 days.

## 4. Conclusions and Future Perspectives

In summary, the preparation strategies and wide applications, as well as the advantages of polymer vesicles in the antimicrobial field, have been concluded. Typically, polymer vesicles usually exhibited superior antimicrobial activity compared with their linear counterparts or free antibiotics due to the enhanced local charge density or high delivery efficiency. Specific functionalities could be achieved by encapsulating functional cargoes while maintaining the antimicrobial activity of polymer vesicles, which is of great significance since bacterial infections are often generated accompanied by different diseases.

To summarize the design strategies and preparation methods of antibacterial vesicles, using positively charged polymers or polypeptides as building blocks is a promising method to endow the polymer vesicles with intrinsic antimicrobial activity and reduced cytotoxicity. However, the mechanism of the high selectivity of antimicrobial vesicles toward bacteria and mammalian cells is still unclear. Moreover, the efficient delivery of antimicrobial agents such as AMPs, antibiotics, and silver nanoparticles is another effective strategy to combat bacteria, taking advantage of the hydrophilic and hydrophobic domains of polymer vesicles. Compared with the unloaded analogs, the use of polymer vesicles as carriers ensure the transportation of those antimicrobial agents to the targeting sites and prevents the reduction of effective dosage due to the defense mechanism of bacteria, such as efflux pump and hydrolysis enzymes. Benefiting from the synergy effect of different antimicrobial agents, the multi-components antimicrobial polymer vesicle systems have shown potentials in combating MDR bacteria. However, despite the significant potentials of antimicrobial polymer vesicles in wide applications including anti-biofilms, wound healing, and tissue engineering, this field is still in a phase of rapid growth and discovery. Therefore, we believe that there are many challenges we should be concerned with in the future.

(1) How to improve the loading content of antimicrobial active components? The antimicrobial agents such as antibiotics are encapsulated in the cavity or membrane of polymer vesicles during self-assembly. This method limits the loading content of antibiotics. To improve the concentration of antibiotics is very important to ensure the entire elimination of bacteria, preventing the generation of drug resistance. Introduction of non-covalent interactions such as π−π interaction, hydrogen bonding, etc. between antibiotics and polymer vesicles might be an effective method to significantly improve the loading content of antibiotics.
(2) How to unleash the advantages of polymer vesicles in combating MDR bacteria? MDR bacteria such as MRSA have threatened the life safety of human beings. Taking advantage of the multifunctional regions of polymer vesicles, different antimicrobial agents including antibiotics, silver nanoparticles, and AMPs could be loaded simultaneously. The synergy effect between those antimicrobial agents might exhibit unexpected antimicrobial activity to MDR bacteria.
(3) How to increase the selectivity toward bacteria and mammalian cells? Antimicrobial agents to kill bacteria by physical interactions with the cell membrane of bacteria such as AMPs and positively charged polymeric nanostructure often show high cytotoxicity to mammalian cells. Shielding of the positive charges before targeting bacteria is the key to increase selectivity. The on-demand release of antimicrobial agents or exposure of the positively charged surfaces of polymer vesicles in response to the bacterial stimulus, such as bacterial toxins or external environmental changes such as pH, concentration of glucose, and so forth is believed to be a feasible strategy. Besides, the decoration of signal molecules on the surface of vesicles to target the cell membrane of bacteria is another alternative.
(4) How to achieve antibacterial and anticancer simultaneously? Considering that the tumor site is commonly accompanied with the bacterial infections due to the decrease of resistance of patients, the simultaneous realization of antibacterial and anticancer is of special significance. One option is to use polymer vesicles with intrinsic antimicrobial activity as “armed” drug carriers, while the other is to take advantage of the high cytotoxicity of antimicrobial agents such as AMPs to kill cancer cells. The synergy between antimicrobial agents and anticancer drugs may bring new insight into the field of cancer treatment.

## Data Availability

The data presented in this study are available on request from the corresponding author.
